# To Prevent Stains from Tincture of Iodine

**Published:** 1872-10

**Authors:** 


### To Prevent Stains from Tincture of Iodine.

It is stated that a few drops of carbolic acid added to an ounce of tincture of iodine will prevent the unsightly stains left by the latter. The effect of the iodine is not impaired.

